# MZB1 expression levels are associated with long-term maintenance of high anti-HBs titers: single-cell RNA-seq with validation by nested case-control and intervention studies

**DOI:** 10.3389/fimmu.2026.1913957

**Published:** 2026-09-02

**Authors:** Mei-Lin Huang, Zhi-Hua Jiang, Li-Ping Hu, Xue-Yan Wang, Qin-Yan Chen, Lu-Juan Zhang, Pei-Yu Wang, Yu-Bi Huang, Xue Hu, Sha Li, Ning Zhang, Tim J. Harrison, Ge Zhong, Zhong-Liao Fang

**Affiliations:** 1 Guangxi Zhuang Autonomous Region Center for Disease Prevention and Control, Guangxi Key Laboratory for the Prevention and Control of Viral Hepatitis, Nanning, China; 2 School of Preclinical Medicine, Guangxi Medical University, Nanning, China; 3 NingMing County Center for Disease Prevention and Control, Ningming, China; 4 Division of Medicine, University College London Medical School, London, United Kingdom

**Keywords:** anti-HBs titers, hepatitis B vaccine, maintenance, MZB1 gene, vaccination, response

## Abstract

**Background:**

Approximately 5–10% of healthy, immunocompetent individuals are low- or non-responders to the HepB vaccine. The mechanism remains obscured. Among responders, immunity may wane over time, becoming undetectable in some. However, in some vaccinees, high anti-HBs titers persist for decades. Investigating the underlying causes may in turn provide clues to solving low- or non-response.

**Methods:**

Single-cell RNA sequencing of blood cells was performed on ten subjects maintaining high anti-HBs titers (>1000 mIU/ml) for over 30 years post-vaccination and ten subjects who had seroconverted. The validation included a nested case-control study (n=102) and an interventional study (n=79). Subjects in the latter study received the HepB vaccine (20 μg) according to a 0-1–6 schedule. Blood samples were collected pre-vaccination, first-and -third dose.

**Results:**

MZB1 expression was significantly upregulated in the B cells of subjects with high anti-HBs titers (P.adj=2.1e-12). Subjects with long-term maintenance of high anti-HBs titers had significantly higher serum MZB1 concentrations (7.1 ± 2.2 ng/mL) than anti-HBs-negative subjects (0.96 ± 0.1 ng/mL) (p < 0.0001). High concentration of MZB1 correlated strongly with a high titer of anti-HBs (r=0.892). The HepB vaccine induces MZB1 prior to anti-HBs. Among individuals who only achieved anti-HBs positivity after the third dose, MZB1 concentrations were already significantly elevated following the first dose (p=0.01) despite still being negative for anti-HBs at that point. The higher the concentration of serum MZB1 protein rises, the better the response to HepB vaccine. The pre-vaccination MZB1 concentrations of non-responders did not increase significantly post-third dose (p=0.548).

**Conclusions:**

Upregulated expression of MZB1 is associated with long-term maintenance of high anti-HBs titers. Serum MZB1 levels are associated with the magnitude of responses to the HepB vaccine, suggesting that serum MZB1 is a valuable predictive biomarker for anti-HBs responses. Further investigation into MZB1 and its upstream regulatory pathways may inform the development of novel adjuvants aimed at achieving sustained antibody titers, which could potentially offer insights into overcoming low or non-responses.

## Introduction

Immunization with HepB vaccine is the most effective approach to control and prevent acute and chronic hepatitis B, including subsequent cirrhosis and HCC (1). Long-term effectiveness of universal vaccination with HepB vaccines has been achieved; globally, the prevalence of persistent HBV infection among children below five years of age fell from 4·7% to 1·3% over two decades (2). Universal vaccination of newborns has reduced the incidence of HCC in Taiwan (3) and elsewhere. A remarkable reduction in HBV-related diseases was observed in Italy of universal vaccination (4).

However, the immune responses to HepB vaccination vary greatly among individuals, with 5-10% of healthy immunocompetent subjects failing to produce protective titers of antibodies (5). Furthermore, the titers of anti-HBs in vaccinees decline over time, becoming undetectable in some, and it has been reported that some vaccinees later become persistently infected, despite being positive for anti-HBs after immunization against HBV at birth (6–8). On the contrary, high titers of anti-HBs may persist in some vaccinees for decades after vaccination (9, 10).

Many studies have investigated the risk factors and mechanisms of low- or non-response to the HepB vaccine (11). The issues remain unsolved (12). Some of them yielded contrasting results. They usually compared subjects who did not develop an immune response with those who developed a high immune response to the hep B vaccine (13, 14).

It has been reported that long-term immunity is provided by memory B cells and T cells in the blood and lymph nodes, as well as long-lived plasma cells and memory T cells in the bone marrow (15). It is unclear how the single-cell landscape of immune cells differs between individuals vaccinated at birth who maintain a high titer of anti-HBs for a long time and those who become rapidly anti-HBs negative.

Investigating the underlying causes may in turn provide clues to solving non-response and breakthrough infection. Here, based on the Long An cohort, Guangxi, China (16), we used single-cell RNA sequencing (scRNA-seq) (17) and a validation study to address these questions.

## Materials and methods

### Study subjects

The study subjects for scRNA-seq were recruited from the Long An cohort. They born between 1987 and 1993 and vaccinated at birth with three 10 μg doses of plasma-derived vaccine. All subjects enrolled in this study were healthy individuals with no known severe comorbidities, chronic inflammatory conditions, or ongoing active diseases. The subjects who maintained a high titer of anti-HBs (>1000 mIU/ml) for at least 30 years was selected as cases while those who became negative for anti-HBs (<10 mIU/ml) were selected as controls. Each subjects provided a 15 mL sample of blood for testing for serological markers of HBV and for scRNA-seq.

For validation, a nested case-control study was performed on serum samples were collected from individuals who maintain high titers of anti-HBs (>1000 mIU/ml) and individuals who became negative for anti-HBs, again from the Long An cohort (9).

For further validation, an interventional study was performed in NingMing county, Guangxi. Study subjects who had not been vaccinated with HepB vaccine were recruited. They were all negative for anti-HBs and were vaccinated with three doses of 20 μg CHO HepB vaccine (NCPC Jintan Bio-Technology Co., Ltd, HeBei, China). Five mL samples of blood from pre-, post-first and -third doses were drawn from each study subject.

Informed consent in writing was obtained from each individual. The study protocol conforms to the ethical guidelines of the 1975 Helsinki Declaration and has been approved by the Guangxi Institutional Review Board.

### Qualitative assays of HBV serological markers

Sera were tested for HBV serological markers using enzyme immunoassays (WANTAI BioPharm, Beijing, China). Quality control for the measurements was performed in accordance with the protocols provided by the manufacturer.

### Quantitative assays of anti-HBs

Serum anti-HBs concentrations were quantified by the Maccura i6000, using HBsAb Quantitative Kits (Maccura Biotechnology Co., Ltd, Chendu, China). According to the protocols provided by the manufacturer, positive and negative cutoffs were calculated with the positive and negative controls. The dynamic range of the kit for anti-HBs is 4 mIU/mL~1000 mIU/mL.

### Collection of peripheral blood mononuclear cells

Two mL of peripheral blood samples were collected into EDTA anticoagulation tubes and processed immediately. PBMCs were separated by density gradient centrifugation using Ficoll-Paque PREMIUM 1.084 g/L sterile solution (GE Healthcare, Uppsala, Sweden). Then, 90% Fetal Bovine Serum + 10% Dimethyl sulfoxide was added to the cryopreservation tube. The cells were stored at −80°C prior to analysis.

### Construction of libraries and single-cell RNA sequencing

Single-cell mRNA libraries were constructed using the DNBelab C Series Single Cell RNA Library Preparation Kit (MGI, Shenzhen, China) according to the protocol provided by the manufacturer. The water-in-oil droplets were prepared by adding single-cell suspension, oil, and beads to a slide using the DNBelab C-TaiM instrument, completing mRNA capture and reverse transcription to generate cDNA. After demulsification, the cDNA was amplified, fragmented, end-repaired, A-tailed, adaptor-ligated, and PCR-amplified to construct the library; the oligo product was amplified, indexed, and purified. Both were denatured into single strands and circularized, then amplified by rolling circle amplification to form DNA nanoballs (DNBs), which were finally sequenced using combinatorial probe-anchor synthesis technology.

### Quality control and determination of the major cell types in the single-cell data

The raw sequencing data were analysed to generate a gene expression matrix using DNBC4tools (version v2.1.1). Subsequent downstream analysis was conducted using the R package Seurat (version 5.3.0) (18). The quality control of cells was carried out, according to the criteria: 1) Cells with fewer than 200 genes identified or more than 90% of the maximum gene count were filtered out. 2) Cells were sorted in descending order based on the proportion of mitochondrial reads, and the top 15% were filtered out. Doublets were identified and removed using DoubletDetection (19). Cell types were annotated by integrating markers from public databases (e.g., CellMarker, PanglaoDB, Cell taxonomy). Differential expression gene (DEG) between groups was determined using the FindMarkers function (test.use = MAST).

### Functional analysis of marker genes or differential genes

GO and KEGG enrichment analyses were performed on differentially expressed genes using the org.Hs.eg.db (version 3.20.0) and clusterProfiler (version 4.14.6) packages in R (version 4.4.1). For KEGG analysis, the organism was set to “hsa” (human). Enriched terms/pathways with a p.adjust <0.05 (Benjamini-Hochberg correction) were considered significant.

### Developmental trajectory analysis

Pseudotime analysis was performed using the Monocle 3 package (version 1.3.7) to reconstruct cellular trajectories and reveal dynamic changes during differentiation (20).

### Gene set enrichment analysis analysis

GSEA was performed with the GSEA function of clusterProfiler (version 4.14.6) by ranking genes using the avg_log2FC from differential expression analysis. Gene sets were obtained from the MSigDB database (C2, C5, H) using the msigdbr package (version 25.1.0). The significance threshold was set at p.adjust <0.05.

### Measurement of serum MZB1 protein

The concentration of serum MZB1 protein was measured using Human Marginal Zone B-and B1-cell-specific Protein (MZB1) Enzyme-Linked Immunosorbent Assa Kit (Bioswamp Co., Ltd, Wuhan, China). According to the protocols provided by the manufacturer, positive and negative cutoffs were calculated with the positive and negative controls. The dynamic range of the kit is 0.125 ng/mL~10 ng/mL and the sensitivity is 0.025 ng/mL.

### Statistical analyses

The Kolmogorov-Smirnov (K-S) test was used to assess overall differences in the distribution of pseudotime values between groups, focusing on shifts in the cumulative distribution. Continuous variables are expressed as means (SD). Categorical data were evaluated using the χ²test or Fisher’s exact test. For quantitative data, the independent samples t-test or Welch’s t-test (for unequal variances) was adopted for normally distributed independent data and the Mann-Whitney U test was used for non-normally distributed independent data. The paired t-test was used for normally distributed, paired data and the paired Wilcoxon signed-rank test was used for non-normally distributed paired data. Bonferroni correction was performed for multiple pairwise comparisons within and across subgroups to control type I errors. Correlation analysis used the Pearson test or Spearman test, depending on the distribution of the data. All P values are two-tailed, and P <0.05 is considered to be significant. R (version 4.4.1) and SPSS software (version 16.0; Chicago, IL, USA) were used for statistical analyses.

## Results

### General characteristics of the study subjects

**Figure 1:**
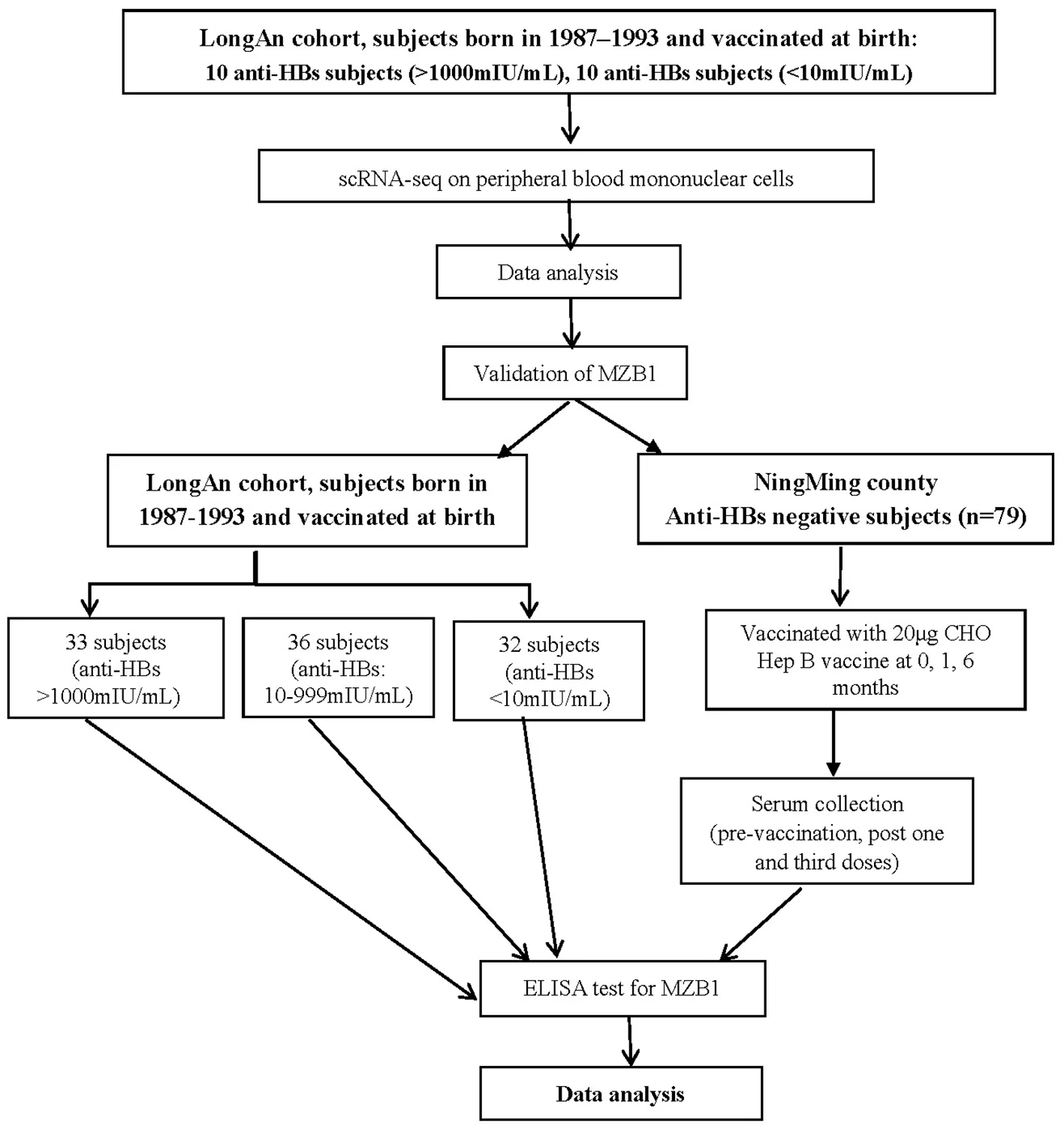
Flow chart of the study participants and their test results.

Twenty subjects were recruited for scRNA-seq analysis, ten of them (seven males, three females) in the case group and another ten (seven males, three females) in the control group (**Table 1**). The positive rate of anti-HBc (7/10, 70%) in the case group does not differ significantly from the control group (5/10, 50%) (Fisher’s exact test, p= 0.6499). For the nested case-control study, 102 study subjects included 62 males and 40 females. The average age was 34.3 ± 1.8 years (range: 30-37). In the interventional study, 79 study subjects were recruited, including 36 males and 43 females. The average age was 42.0 ± 9.9 years (range: 25-68) (**Table 2**; **Figure 1**).

**Table 1:** Baseline information of the study subjects for single-cell RNA sequencing.

Code	Group	Sex	Age	Anti-HBs status			
				2005	2009	2015	2024
09LA1142	Control	M*	37	+	–	–	0
09LA1232	Control	M	34	+	–	–	0
ACS025	Control	F#	34	+	–	–	0
ADJ075	Case	M	34	+	+	>1000 mIU/mL	>1000 mIU/mL
ADK087	Case	F	36	+	+	>1000 mIU/mL	>1000 mIU/mL
ADK122	Case	M	37	+	+	>1000 mIU/mL	>1000 mIU/mL
AGG102	Case	M	36	+	+	>1000 mIU/mL	>1000 mIU/mL
AGY059	Case	M	31	+	+	>1000 mIU/mL	>1000 mIU/mL
ANF082	Control	M	37	+	–	–	0
ANF175	Control	F	36	+	+	–	0
ANL070	Control	M	36	+	–	–	0
ANS040	Control	M	36	+	–	–	0
ANS050	Case	F	36	+	+	>1000 mIU/mL	>1000 mIU/mL
ANS124	Control	M	35	+	+	–	0
ANS385	Control	M	31	+	–	–	0
ANT046	Control	F	36	+	–	–	0
ATW029	Case	M	33	+	+	>1000 mIU/mL	>1000 mIU/mL
ATW057	Case	F	33	+	+	>1000 mIU/mL	>1000 mIU/mL
ATX266	Case	M	36	+	+	>1000 mIU/mL	>1000 mIU/mL
ATZ080	Case	M	35	+	+	>1000 mIU/mL	>1000 mIU/mL

*M: Male, #F: Female.

**Table 2:** The concentration of serum MZB1of the study subjects in the validation study.

	LongAn county Vaccinated at birth group							Notes
Study Subjects (n=102, 62 males, 40 females)	32 anti-HBs (–) subjects		36 subjects, anti-HBs: 10-999mIU/mL			33 subjects, anti-HBs: ≥1000mIU/mL		
MZB1* concentration (ng/mL)	0.96 ± 0.1#		2.6 ± 1.9			7.1 ± 2.2		K-W Htest χ² =77.26, df=2, All p value among the three levels of MZB1 was 0.0001

NingMing conuty Never vaccinated against HBV								
	Pre-vaccination	Post-first dose				Post-third dose		
Study subjects (n=79, 36 male, 43 female)	79 subjects A^$^: 5 subjects B ^&^: 38 subjects	43 anti-HBs (–) subjects		27 subjects, anti-HBs: 10-999mIU/mL	9 anti-HBs ≥1000mIU/mL subjects	5 anti-HBs (–) subjects	28 subjects, anti-HBs: 10-999mIU/mL	46 subjects anti-HBs ≥1000mIU/mL
MZB1 concentration (ng/mL)	0.83 ± 0.3^@^ A0: 0.77 ± 0.6 B0: 0.83± 0.3	1.11 ± 0.8^¥^ A1: 0.69 ± 0.3 B1: 1.17± 0.8		2.69 ± 2.4	9.33 ± 4.6	A3: 1.02 ± 0.4	4.81 ± 2.7	7.44 ± 2.9

*: MZB1, marginal zone B and B1 cell specific protein. #: mean ± standard deviation (SD). @: total MZB1 concentration in the 79 subjects. ¥: total MZB1 concentration in the 43 subjects. $: ‘A’ represents s subjects who were non-responders post-third. &: ‘B’ represents subjects who were negative for anti-HBs post-first dose but became positive post-third dose. A0, A1, A3, B0, and B1 represent pre-vaccination, post-first and -third dose, respectively.

Statistical comparisons: A0 vs. A1: p=0.805; A0 vs. A3: p=0.474; A1 vs. A3: p=0.221; B0 vs. B1: p=0.003.

### Single cell atlas of PBMCs from subjects with high titers of anti-HBs and from anti-HBs negative subjects

**Figure 2:**
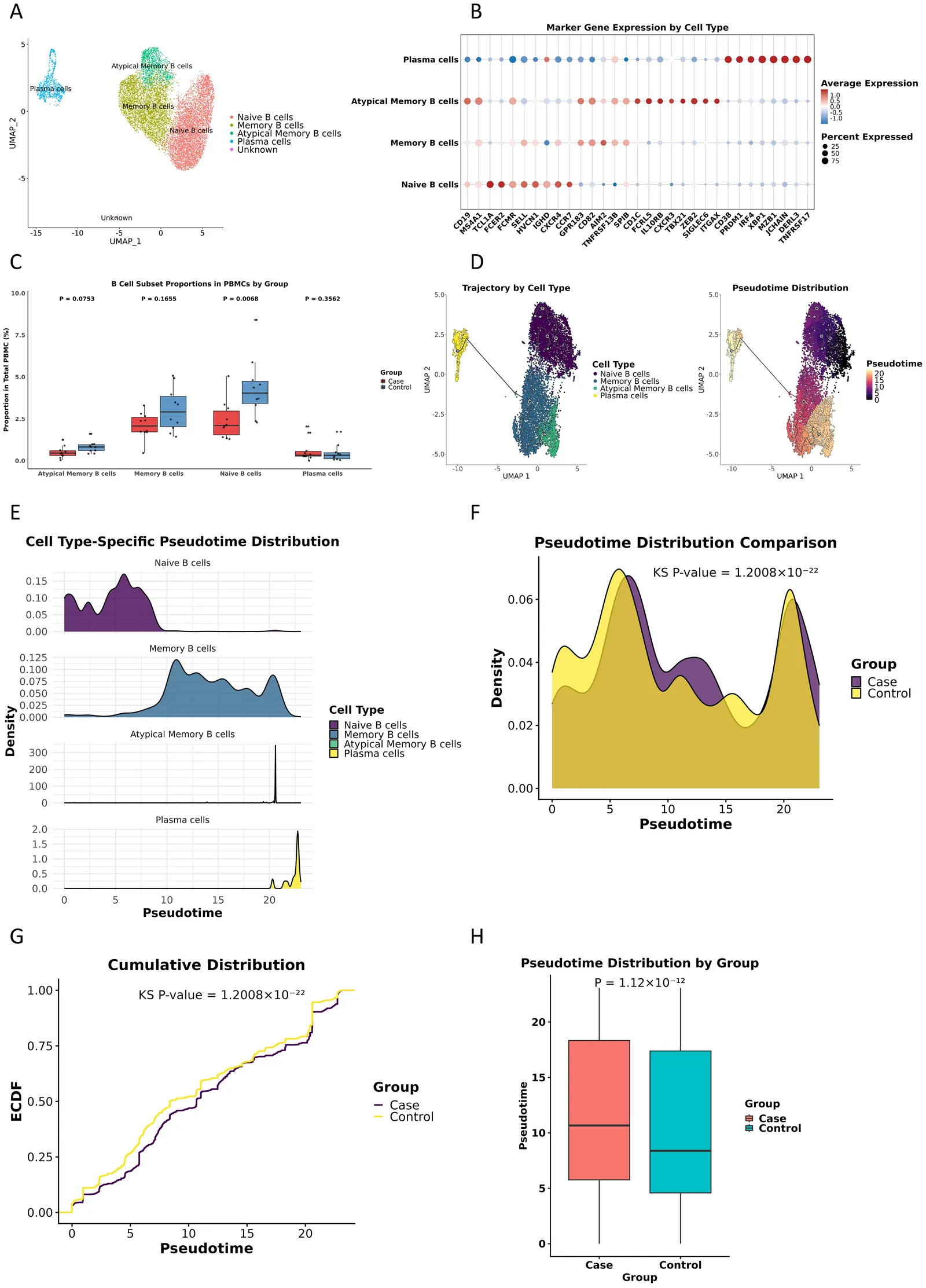
Single-cell transcriptome map of PBMCs from adults vaccinated against HBV at birth. **(A)** UMAP plot of the 162,172 single cells from 20 individuals, revealing 23 distinct cell clusters. Each colour represents a cluster, and each point represents a single cell. **(B)** Plot visualizing the distribution of the 13 identified peripheral blood cell lineages. Each colour represents a different cell type, and each point represents a single cell. **(C)** Dot plots showing the expression of the key marker genes (x-axis) of major cell types (y-axis). The depth of the colour from light to dark and the sizes of the dots represent the average expression from low to high and the percentage of cells expressing the gene. **(D)** Bar plot of cell type proportions per sample in the case and control groups, coloured by cell types. **(E)** Box plot of cell type proportions in the case and control groups, the x-axis represents different cell types, and the y-axis represents the proportion. The boxes are coloured by groups (Wilcoxon test with Benjamini–Hochberg correction).

After single-cell RNA sequencing and quality control and filtering, a total of 162,172 cells were finally obtained, including 82,104 cells from case group and 80,068 cells from control group. Twenty-three clusters were identified (**Figure 2A**). According to the expression of canonical marker genes, 13 cell types were identified (**Figure 2B, C**), including B cells, plasma cells, CD4+ T cells, CD8+ T cells, MAIT, proliferative cells, NK cells, CD16+ monocytes, CD14+ monocytes, cDC, pDC, neutrophils, and platelets. The cell type distribution across individual samples is presented in **Figure 2D**. The proportion of B cells in the total of PBMCs in the case group was significantly lower than in the control group (Wilcoxon test, adjusted p=0.0039, **Figure 2E**).

### Single-cell landscape of B cells and plasma cells

**Figure 3:**
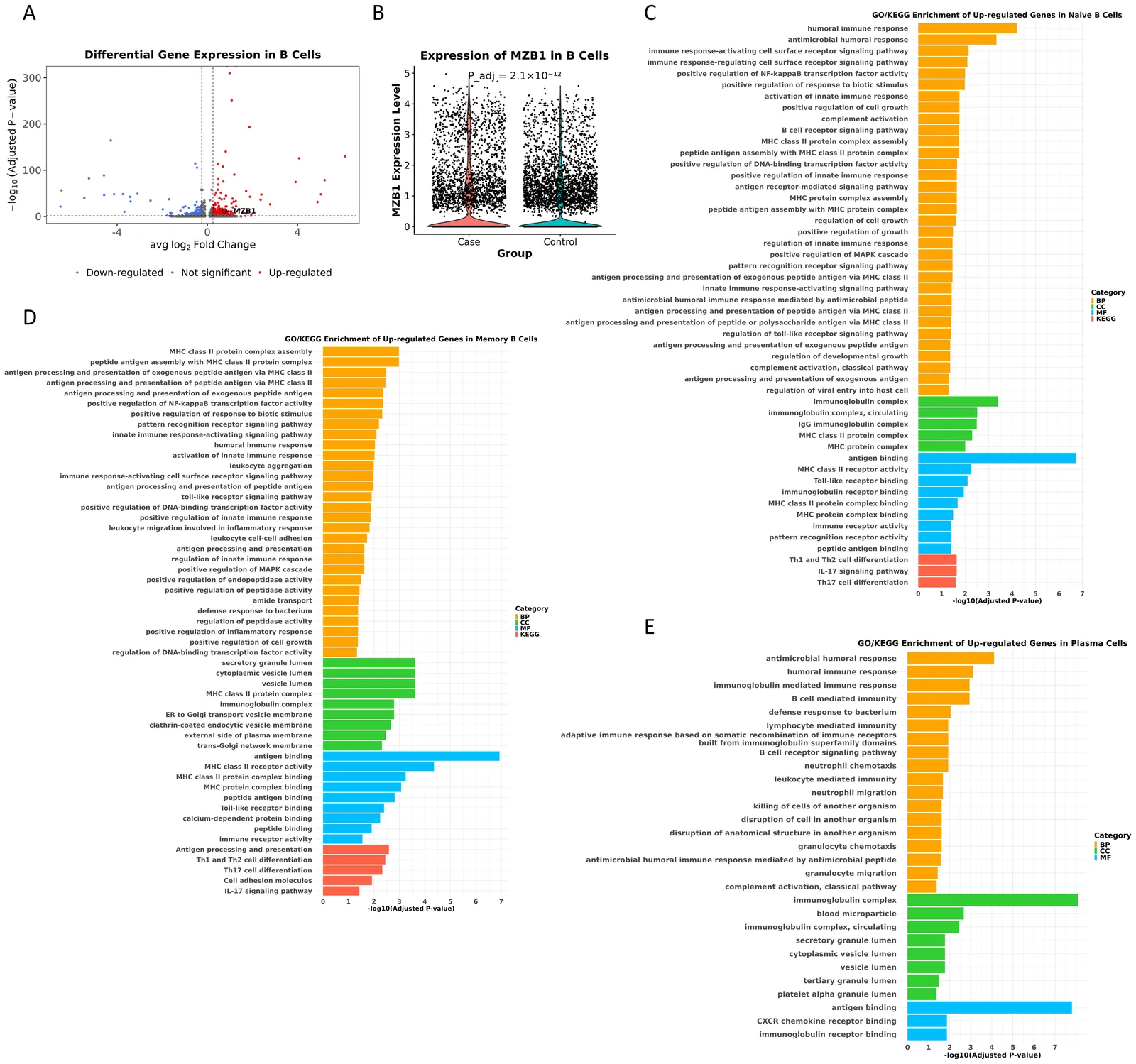
Single-cell transcriptome map of B cells. **(A)** UMAP visualization of B cell subsets in the case and control groups. **(B)** Dot plot of marker genes for B cell subsets. The depth of the colour and the sizes of the dots represent the average expression levels and the percentage of cells expressing the gene. **(C)** Box plot of the proportion of B cell subsets in the total PBMCs for the case and control groups (Wilcoxon test with Benjamini–Hochberg correction). **(D)** The left panel shows the developmental trajectory of B cells, with distinct colours representing different cell types. The right panel illustrates the pseudotime distribution, where the colour gradient from black to yellow indicates the progression from early to late stages in cell development. **(E)** The pseudotime density distribution across various B cell types. **(F)** Density plot comparing the pseudotime distributions of B cells between the case and control groups, which exhibits distinct peaks ((Kolmogorov–Smirnov (KS) test)). **(G)** Compares the cumulative distributions of pseudotime for the case and control groups. The x-axis represents pseudotime, while the y-axis shows the cumulative distribution function (ECDF) (KS test). **(H)** Box plot of pseudotime values for B Cells in the case and control groups (Wilcoxon test with Benjamini–Hochberg correction).

Subsets of B cells and plasma cells were extracted from PBMCs for the secondary clustering analysis. Four subsets of B cells were identified according to marker genes (**Figure 3A, B**), including naive B cells, memory B cells, atypical memory B cells and plasma cells. The proportion of naive B cells was significantly lower in the case group (p=0.0068, **Figure 3C**).

The results of pseudotime analysis showed that naive B cells, memory B cells, and plasma cells are sequentially distributed along the pseudotime axis and form a continuously trajectory in the low-dimensional space (**Figure 3D**), suggesting that this pseudotime sequence may reflect the primary path of differentiation from naive B cells through the memory stage to plasma cells (**Figure 3E**). The density of B cells in the case group is significantly lower than the control group in the early pseudotime state, while it is higher than that in control group in the late state (**Figure 3F**). The Empirical Cumulative Distribution Function curves of the pseudotime values of B cells showed that B cells in the case group differentiate and mature more rapidly (**Figure 3G**); there are more cells in the high pseudo-time stage. The data also showed that the pseudotime values of B cells were significantly higher in the case group than the control group, suggesting that B cells in the case group have developed/differentiated to a later and more mature stage (**Figure 3H**).

### Upregulated expression of the MZB1 gene is associated with maintenance of long-term, high titers of anti-HBs

**Figure 4:**
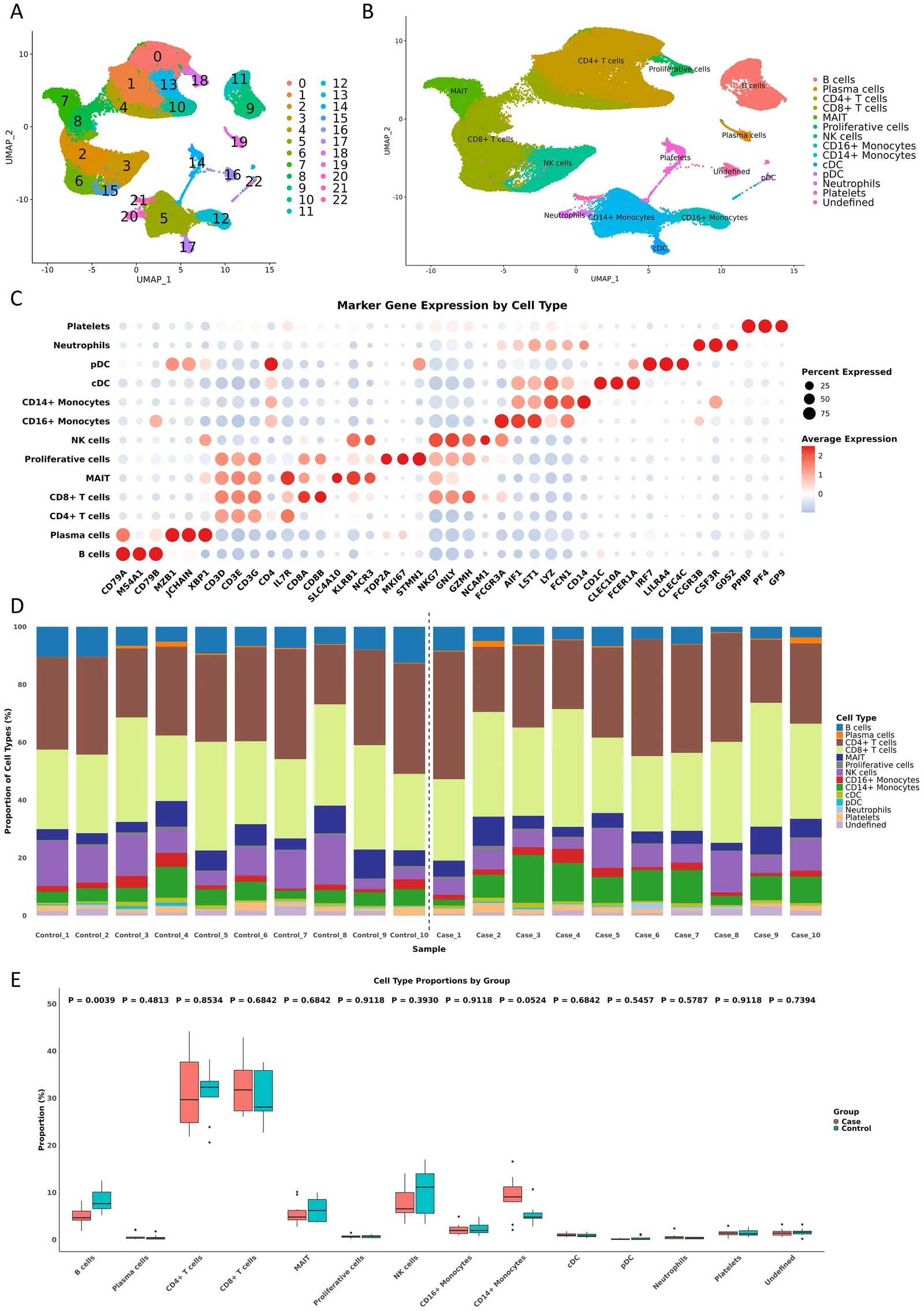
Differential gene expression (DGE) and functional enrichment. **(A)** Volcano plot of the MZB1 gene in differentially expressed genes in B cells, between the case and control groups. MZB1 is marked and annotated. **(B)** Expression profile of the MZB1 gene in B cells from the case and control groups, each dot represents a single cell (Wilcoxon test with Benjamini–Hochberg correction). **(C)** GO and KEGG analysis results of up-regulated genes in naive B cells from the case group, coloured by different categories. **(D)** GO and KEGG analysis results of up-regulated genes in memory B cells from the case group, coloured by different categories. **(E)** GO and KEGG analysis results of up-regulated genes in plasma cells from the case group, coloured by different categories.

Differential gene expression analysis was performed for B cells and the subsets naive B cells, memory B cells and plasma cells. We identified 151 upregulated genes in all B cells in the case group. The expression levels of marginal zone B and B1 cell specific gene (MZB1) were significantly upregulated in all B cells (P.adj=2.1e-12, **Figure 4A, B**), although its upregulated expression in each subset of B cells did not reach significance, suggesting that upregulated expression of the MZB1 gene is associated with maintenance of long-term, high titers of anti-HBs.

Forty-one, 62 and 39 upregulated DEGs were obtained in naive B cells, memory B cells and plasma cells, respectively. The results of functional enrichment of these upregulated DEGs using GO and KEGG pathway analysis showed that all three cell types were significantly enriched in the pathways of antigen binding and humoral immune response. Plasma cells were enriched in more pathways. Innate immunity regulation and antigen presentation and processing related pathways are prominent in memory B cells and naive B cells (**Figures 4C–E**).

### Gene set enrichment analysis

**Figure 5:**
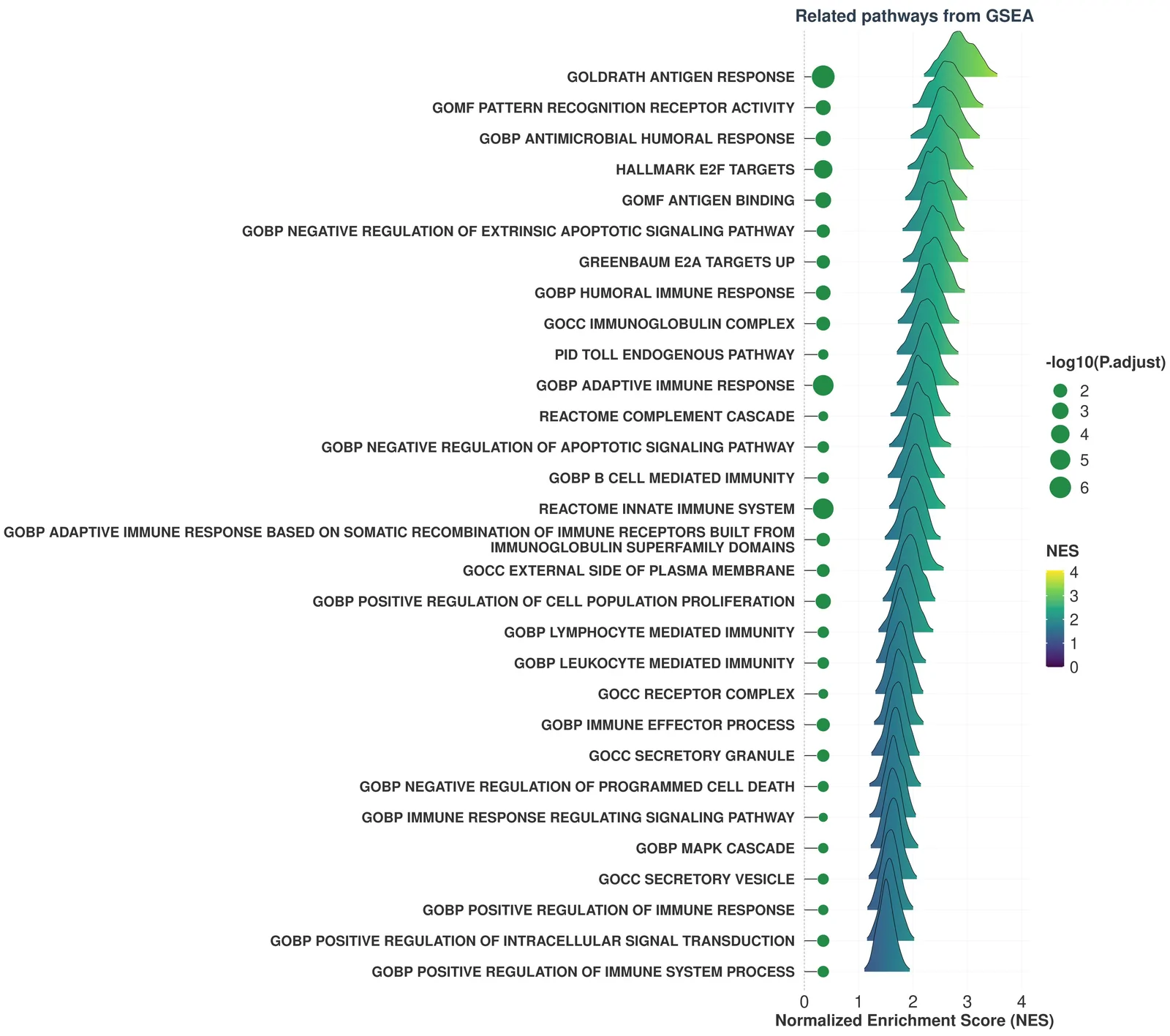
Gene set enrichment analysis (GSEA) pathways enriched by differentially expressed genes. The ridge plot shows the GSEA results for up-regulated genes in B cells from the case group. The x-axis indicates the normalized enrichment score (NES). The y-axis lists pathways, ordered by NES. Circle sizes reflect enrichment significance (-log10 (P. adjust)). The colour of the ridge represents the magnitude of the NES.

GSEA was performed on B cells to identify enriched gene sets in the case and control groups. Several major categories of significantly enriched gene sets related to the long-term maintenance of high antibody titers were identified (**Figure 5**). The pathways from the figure suggest that B cells in the case group exhibited an enhanced activation status in these biological processes.

### High concentrations of serum MZB1 protein were associated with maintaining long-term high titers of anti-HBs

In the nested case-control study, the average concentration of serum MZB1 protein did not differ significantly between males (3.6 ± 3.1) ng/mL) and females (3.4 ± 3.1) ng/mL (p=0.544), suggesting that sex did not influence the concentration of serum MZB1. We found that a high concentration of MZB1 correlated strongly with a high titer of anti-HBs (r=0.892). The average concentrations of serum MZB1 protein in subjects who maintained a high titer of anti-HBs (>1000 mIU/ml) for over 30 years post-vaccination was significantly higher than that of subjects who were negative for anti-HBs (p=0.0001). We also found that the average concentrations of serum MZB1 protein in subjects with anti-HBs titers of 10-999mIU/mL was significantly lower than that of high anti-HBs subjects (p=0.0001) but significantly higher than that of subjects who were negative for anti-HBs (p=0.0001). These results confirmed the findings found in scRNA-seq and suggested that high expression of MZB1 is a valuable biomarker to predict the long-term maintenance of high titers of anti-HBs (**Table 2**).

### The higher the concentration of serum MZB1 protein rises, the better the response to the hepatitis B vaccine

In the interventional study, the positive rates of anti-HBs post-first and -third dose vaccinations were 45.6% (36/79) and 93.7% (74/79), respectively. The mean of anti-HBs titres post-first and -third dose vaccinations was 374.8 and 699.6 mIU/mL, respectively. Compared to pre-vaccination (0.83± 0.3 ng/mL), the concentrations of MZB1 post-first dose (4.4± 4.2 ng/mL) (adjusted p=0.001) and -third dose (6.4± 3.1 ng/mL) (adjusted p=0.001) rose significantly. The concentration of MZB1 post-third dose was also significantly higher than post-first dose (p=0.0001). The concentrations of MZB1 differed significantly between those who became positive and those who remained negative for anti-HBs post-vaccination (adjusted p=0.001) (**Table 2**). These data suggested that the higher the concentration of serum MZB1 protein rises, the better the response to HepB vaccine.

### Temporal precedence of MZB1 protein induction, relative to high titers of anti-HBs

There are 38 subjects whose titers of anti-HBs post-first dose were negative. We found that their concentrations of MZB1 post-first dose were significantly higher than pre-vaccination (adjusted p=0.003). The titers of anti-HBs in 21 of them rose to >1000 mIU/ml post-third dose, suggesting that HepB vaccine induces MZB1 prior to anti-HBs and demonstrating a clear temporal precedence that supports MZB1 as a predictive marker for the subsequent titer of anti-HBs (**Table 2**).

### Exploratory analysis of serum MZB1 levels in non-responders

There are five subjects of the 79 subjects who remained negative for anti-HBs post-third dose. Their concentrations of MZB1 post-first (t=0.25, p=0.805) and -third doses (t=-0.75, p=0.474) had not increased significantly compared to pre-vaccination (p=0.841, p=0.548, respectively) (**Table 2**), suggesting that a low concentration of MZB1 is associated with non-response to HepB vaccine.

## Discussion

Through this study, we are the first to explore factors associated with long-term maintenance of high anti-HBs titers using transcriptional profiles of immune memory cells from peripheral blood of HepB vaccine recipients. The major findings are that upregulated expression of the MZB1 gene in B cells is associated with long-term maintenance of high titers of anti-HBs and its concentration in serum correlated strongly with anti-HBs titers. Our data suggest that MZB1 stands as a promising predictive biomarker for the persistence of anti-HBs responses, although further direct functional validation is required to fully decouple causality from general B-cell differentiation. The higher the concentration of serum MZB1 protein rises, the better the response to HepB vaccine. Additionally, B cells from individuals with high anti-HBs titers exhibited a more mature differentiation state and enhanced activation status. The strength of this study is that the findings from single-cell RNA sequencing were confirmed by both nested case-control and interventional studies.

When a vaccine is administered, the antigen is first taken up by dendritic cells (DCs). Naive T cells recognize the antigen displayed by DCs and mature. Mature DCs activate CD4+ T cells, leading to their differentiation into follicular helper T cells (Tfhs). Tfhs encourage B-cell differentiation into antigen-specific B cells, such as memory B cells and plasma cells (21). Long-term immunity is provided by memory B cells, memory T cells and long-lived plasma cells (22–24). With enzyme‐linked immunosorbent spot (ELISPOT) assay, one study reported that some cytokines, such as CD40L, Interleukin 2, Interferon Gamma and Tumor Necrosis Factor Alpha, are associated with maintenance of anti-HBs titers (25). However, they evaluated cytokines and immune cells before and after a booster dose of vaccine. Therefore, their results may not answer our question. In our study, we are the first to compare the transcriptional profiles of immune cells between subjects who maintained high titers of anti-HBs for at least 37 years and others who became anti-HBs negative. Therefore, our results may address better the factors associated with long-term antibody persistence.

The contribution of HB vaccination to the control of HBV infection and reduction of the burden of HBV-related liver disease has been documented (21). However, whether immunization of neonates provides lifelong protection remains contentious (26, 27). Some vaccinees become persistently infected years after anti-HBs seroconversion (6–8, 28). Furthermore, 5-10% of healthy immunocompetent subjects fail to produce protective titers of antibodies (5). A high titer of anti-HBs generally correlates with long-lasting protection from HBV infection (24, 29). Our findings imply for developing novel adjuvants to enhance hepatitis B vaccine efficacy, which may not only improve current vaccines and their immune protection, leading to lifelong immunity, but also resolve low and non-responses.

When a vaccine is injected, vaccine antigen-specific B cell antigen receptor signalling, amplified by T cell help (via CD40L and IL-21), induces transcription factors such as IRF4, XBP1, and Blimp-1, and the latter directly promotes MZB1 gene expression (30–33). MZB1 is an endoplasmic reticulum-localized cochaperone that is highly expressed in marginal-zone and B1 B cells and is strongly upregulated during plasma-cell differentiation, where it promotes efficient immunoglobulin folding and secretion (34). MZB1 has multifaceted roles in health and disease. It regulates endoplasmic reticulum calcium stores and calcium signalling, thereby modulating antibody secretion and B-cell activation thresholds (35). It also promotes integrin activation and adhesion in innate-like B cells through ERp57-dependent effects, supporting the cellular interactions required for rapid humoral responses (35). Genetic deletion or depletion of MZB1 impairs plasma-cell differentiation and reduces antibody titers (30). Although our study cannot perform such functional deletions in human subjects to verify the absolute necessity of MZB1 specifically for HBsAg, the molecular necessity of MZB1, in general, for antibody secretion, established by Andreani et al. (30), strongly supports the biological plausibility of our clinical observations. Our study bridges this fundamental mechanism with long-term, human vaccine translation.

In this study, we found that the HepB vaccine induces MZB1 expression prior to the appearance of anti-HBs, demonstrating a clear temporal association that underscores the potential of MZB1 as a predictive biomarker for high anti-HBs titers. We also observed that B cells from individuals with long-term maintenance of high anti-HBs titers have differentiated into a more mature stage and exhibit an enhanced activation status. In these individuals, MZB1 gene expression was significantly upregulated across all B cells. We hypothesize that the HepB vaccine induces MZB1 expression, which in turn supports antibody synthesis and secretion. Activated B cells produce more MZB1, thereby facilitating continued antibody secretion and maintaining long-term production of high anti-HBs titers.

Many studies have addressed the causes of negative and poor responses to HepB vaccine (36, 37). However, the issue remains unresolved (38). Recently, with single-cell RNA sequencing, Zhao et al. found that defective antigen presentation, decreased T cell activity, and reduced IL-4 secretion are associated with non-response to HepB vaccine (13). In this study, we found that MZB1 levels did not increase post-third dose in vaccine non-responders. However, the extremely limited sample size of non-responders (n=5) precludes drawing robust statistical conclusions. These preliminary observations merely describe a distinct MZB1 kinetic trend in non-responding individuals, which warrants further validation in larger cohorts.

Intriguingly, MZB1 expression did not reach statistical significance within individual B-cell subsets, whereas the pan-B-cell analysis yielded a highly significant difference. This is most likely due to the reduced statistical power that accompanies partitioning of cells into smaller static sub-clusters (39). The distinct bimodal expression pattern observed exclusively in the case group further supports the dynamic and heterogeneous nature of B-cell activation. MZB1 functions as an activation-induced endoplasmic-reticulum chaperone, rather than a static lineage marker (35); upon stimulation, antigen-driven activation occurs heterogeneously, where only a fraction of responding B cells undergoing active differentiation robustly up-regulate MZB1 (40).

Although causality cannot be established from the present data, we propose the following hypotheses: First, as an ER cochaperone that promotes immunoglobulin folding and secretion, sustained MZB1 expression may support the long-term secretory function of anti-HBs-producing plasma cells (30, 35). Second, higher MZB1 levels may facilitate more efficient B-cell differentiation into memory and plasma cells, as suggested by the more advanced pseudotime state observed in those who maintained high antibody titers. Third, the fact that vaccination induces MZB1 before anti-HBs appears raises the possibility of a feed-forward mechanism that helps counteract natural titer waning. However, these hypotheses require confirmation.

Because inadequate MZB1 induction is associated with non-response, and higher MZB1 levels correlate with both stronger primary responses and long-term persistence of antibody titers, strategies that enhance MZB1 expression or its upstream regulators (e.g., the Blimp-1–MZB1 axis) could convert non-responders into responders and prolong protective immunity (30). This provides a rational basis for developing next-generation HepB vaccine adjuvants.

Natural HBV exposure may be an important confounder. However, all scRNA-seq participants were confirmed negative for HBsAg, eliminating active HBV infections. Although historical exposure (indicated by anti-HBc positivity) was detected in both groups due to the regional endemic background, the positivity rates showed no statistically significant difference between the case and control group. Therefore, this potential natural boosting effect was statistically balanced and equally distributed between the two comparison arms, it is highly unlikely to be the primary confounding driver explaining the divergent anti-HBs maintenance profiles. Nevertheless, the lack of continuous, longitudinal monitoring for community exposure during the 30-year span remains an inherent limitation of our study. We acknowledge that HLA genotypes are critical determinants of the HepB vaccine response. Due to the retrospective nature of this long-term follow-up study and the limited DNA availability from the scRNA-seq participants, HLA genotyping was unfortunately not performed. This is a clear limitation of our study.

It is clear from our study that serum MZB1 concentration may be a valuable biomarker for predicting the long-term maintenance of high anti-HBs titers. However, several questions remain to be addressed. First, is there a causal relationship between MZB1 expression and anti-HBs titers? Second, does the known upstream regulatory pathway IRF4/BLIMP-1 → MZB1 (30–33) play a dominant role in the vaccine response? Third, is reduced MZB1 expression a consequence of dysregulation in this regulatory axis, or is it an independent cause?

In conclusion, we demonstrated that upregulated expression of *MZB1* is associated with long-term maintenance of high anti-HBs titres, and its concentration correlates strongly with anti-HBs titers. Serum MZB1 levels are associated with the magnitude of response to the HepB vaccine. The higher the concentration of serum MZB1 protein rises, the better the response to HepB vaccine. These findings demonstrate a strong chronological association and biological plausibility between MZB1 expression and long-term anti-HBs responses and suggest that serum MZB1 is a valuable predictive biomarker for anti-HBs responses. Fully elucidating the role of MZB1 and its upstream regulatory pathways may provide valuable insights for the development of novel adjuvants aimed at achieving lifelong protection and resolving low or non-responses.

## Data Availability

The original contributions presented in the study are publicly available. This data can be found here: [https://www.scidb.cn/s/JrMZJ3]. Further inquiries can be directed to the corresponding authors.
